# Performance of prostate health index and PSA density in a diverse biopsy‐naïve cohort with mpMRI for detecting significant prostate cancer

**DOI:** 10.1002/bco2.91

**Published:** 2021-06-15

**Authors:** Samuel Carbunaru, James Stinson, Rilwan Babajide, Courtney M. P. Hollowell, Ximing Yang, Marin Sekosan, Karen Ferrer, Andre Kajdacsy‐Balla, Josephine Abelleira, Maria Ruden, Patrice King‐Lee, Daniel P. Dalton, David D. Casalino, Rick A. Kittles, Peter H. Gann, Edward M. Schaeffer, Adam B. Murphy

**Affiliations:** ^1^ Department of Urology Northwestern University Feinberg School of Medicine Chicago IL USA; ^2^ Division of Urology Cook County Health and Hospitals System Chicago IL USA; ^3^ Pritzker School of Medicine University of Chicago Chicago IL USA; ^4^ Department of Pathology Northwestern University Feinberg School of Medicine Chicago IL USA; ^5^ Department of Pathology & Laboratory Medicine Cook County Health and Hospitals System Chicago IL USA; ^6^ Department of Pathology University of Illinois at Chicago Chicago IL USA; ^7^ Department of Population Sciences City of Hope Duarte CA USA

**Keywords:** African American, biomarker, cancer detection, PIRADS 3, Prostate Health Index, prostate MRI, PSA density

## Abstract

**Objective:**

To compare Prostate Health Index (PHI) and prostate‐specific antigen (PSA) density as secondary tests after multiparametric magnetic resonance imaging (mpMRI) in improving the detection accuracy of Gleason grade group (GG) 2‐5 prostate cancer (PCa) and in decreasing unnecessary biopsies in a multiethnic biopsy‐naïve population.

**Methods:**

From February 2017 to February 2020, we recruited consecutive biopsy‐naïve men in participating urology clinics for elevated PSA levels. They all had a PHI score, mpMRI, and prostate biopsy. Experienced genitourinary radiologists read all mpMRI studies based on PIRADS version 2.0. Logistic regression models were used to generate receiver operating characteristic curves. Models were tested for effect modification between Race (Black vs White) and both PHI and PSA density, and Race and PIRADS to determine if race impacted their prediction accuracy. Sensitivity, specificity, and predictive values of PHI and PSA density thresholds were calculated by PIRADS scores. The primary outcome was GG2‐5 PCa, that is, Gleason score ≥3 + 4.

**Results:**

The study included 143 men, of which 65 (45.5%) were self‐reported Black. Median age was 62.0 years and 55 men (38.4%) had GG2‐5 PCa. Overall, 18.1% had PIRADS 1‐2, 32.9% had PIRADS 3, and 49.0% had PIRADS 4‐5. For the binary logistic regressions, the interactions between PIRADS and Race (*P* = .08), Log (PHI) and Race (*P* = .17), and Log (PSA density) and Race (*P* = .42) were not statistically significant. Within PIRADS 3 lesions, a PHI ≥49 prevented unnecessary biopsies in 55% of men and missed no GG2‐5 PCa, yielding a negative predictive value of 100%. There was no reliable PHI or PSA density threshold to avoid PCa biopsies in PIRADS 1‐2 or 4‐5.

**Conclusions:**

PHI and PSA density can be used after mpMRI to improve the detection of GG2‐5 PCa in a biopsy‐naïve cohort. PHI may be superior to PSA density in PIRADS 3 lesions by avoiding 55% of unnecessary biopsies. Using both PHI and PSA density in series may further increase specificity and lead to fewer unnecessary biopsies, but further larger studies are warranted to determine the optimal threshold of each biomarker.

## 1 INTRODUCTION

While prostate‐specific antigen (PSA) testing has led to improved detection of prostate cancer (PCa) and a reduction in PCa‐specific mortality,^1^ its poor specificity in detecting clinically significant Gleason grade group (GG) 2‐5 PCa (ie, Gleason score ≥3 + 4) has resulted in over‐detection and overtreatment of indolent PCa.^2^, ^3^ Multiparametric magnetic resonance imaging of the prostate (mpMRI) has emerged as an important tool to enhance the detection of GG2‐5 PCa and guide targeted biopsies while reducing the detection of indolent PCa.^4^, ^5^ However, by itself, it is an imperfect test. The rate of detection of GG2‐5 PCa varies widely, and it may miss up to 24% of clinically significant PCa.^6^, ^7^ Additionally, systematic reviews have shown that the negative predictive value (NPV) of mpMRI ranges from 63% to 98% for GG2‐5 PCa,^8^, ^9^ which may lead to a high number of unnecessary prostate biopsies.^10^ This is especially true in men with equivocal scores on the Prostate Imaging Reporting and Data System (PIRADS). While scores on the higher (PIRADS 4‐5) and lower (PIRADS 1‐2) ends of this scale have been shown to have high positive predictive value (PPV) and NPV, respectively, the prevalence of GG2‐5 in PIRADS 3 lesions ranges between 8% and 47%,^11^, ^12^ making biopsy decisions for patients in this group difficult. Moreover, it is unclear if mpMRI performs differently in non‐White populations since most of the validations do not include large numbers of minorities.

Given mpMRI’s limitations, many have suggested using serum‐based biomarkers, such as PSA density and the Prostate Health Index (PHI), to improve the detection of GG2‐5 PCa and better identify men who can avoid a prostate biopsy. Both PHI and PSA density have been shown to have higher specificity for GG2‐5 PCa than PSA, which could aid in preventing unnecessary biopsies.^13^, ^14^, ^15^, ^16^, ^17^, ^18^ Previous studies have demonstrated that PSA density^12^, ^19^, ^20^, ^21^ and PHI^17^, ^22^ improve the PPV and NPV of mpMRI in detecting GG2‐5 PCa in multivariable models and in series.

Few studies have investigated the effect that race has on these biomarkers. The cohorts previously studied have had minimal representation of Black men, a group with a higher than average incidence of PCa and greater risk of GG2‐5 PCa.^23^ No study to date has investigated whether PHI improves the accuracy of mpMRI in detecting GG2‐5 PCa in biopsy‐naïve men with adequate representation of Black men. The objective of this study was to determine the utility of PHI and PSA density in series after mpMRI in improving the accuracy for detecting GG2‐5 PCa and improving the specificity in a multiethnic population. We additionally focus on the impacts of both markers in PIRADS 1‐2, 3, and 4‐5 lesions.

## 2 METHODS

### 2.1 Patient selection

From February 2017 to February 2020, 143 men who met the selection criteria were referred to participating urology clinics for elevated PSA levels and who underwent mpMRI were recruited into this prospective study. Selection criteria included men between the ages of 40 and 79 years who never had a previous prostate biopsy. Men with a history of a previous prostate cancer were excluded from the study. Additionally, men with signs and symptoms of infection, prostatitis, or who were taking 5‐alpha reductase inhibitors were excluded. Participating urologists performed a digital rectal examination (DRE) on all men at diagnostic biopsy. The DRE was classified as normal or suspicious.

### 2.2 Imaging

All patients underwent mpMRI on 3.0‐T scanners (Skyra or Verio, Siemens Medical System, Erlangen, Germany) with triplanar T2‐weighted, axial dynamic contrast‐enhanced (DCE), diffusion‐weighted imaging (DWI), according to previously designed protocols. These mpMRI studies were blinded and read by two highly experienced genitourinary radiologists (DC and WM) with significant experience in interpreting prostate MRI. Lesions were assigned suspicion scores from 1 to 5 based on the standardized PIRADS criteria version 2.0.^24^ For our analysis, we considered PIRADS 1‐2 lesions negative, PIRADS 3 lesions equivocal, and PIRADS 4‐5 lesions highly suspicious.

### 2.3 Prostate biopsy

Regardless of PIRADS score, all patients underwent MRI‐informed prostate biopsy within 3 months of mpMRI. Patients who had PIRADS 3‐5 lesions underwent MRI‐transrectal ultrasound‐guided fusion biopsy (ie, targeted biopsy and 12‐core systematic biopsy). All PIRADS 1‐2 men underwent systematic transrectal ultrasound‐guided biopsy alone, with 42% of men also receiving a targeted MRI‐guided biopsy. Early in the study from 2017‐2018, PIRADS 1‐2 lesions were also targeted for a prostate biopsy, but this practice was stopped in 2019 as the detection rates were determined to be low. All procedures were done by urologists with several years of experience with transrectal and fusion biopsy using the Invivo UroNav.

### 2.4 Pathologic review

Pathological assessment of biopsy specimens was performed by expert uro‐pathologists (XY, MS, KF, and AKB) in accordance with the 2016 International Society of Urological Pathology Consensus Conference, that is, GG1: Gleason score ≤6; GG2: Gleason score 3 + 4 = 7; GG3: Gleason score 4 + 3 = 7; GG4: Gleason score = 8; and GG5: Gleason score = 9‐10.^25^ The primary outcome was GG2‐5 PCa on biopsy.

### 2.5 PHI and PSA assay

Serum PHI samples were drawn immediately before the biopsy and centrifuged to extract plasma on the day of biopsy in accordance with the manufacturer's recommendations (Beckman Coulter). Blood was collected at least 4 days after any prostate manipulation (eg, DRE). PSA density was derived from the PSA in the PHI assay and the MRI‐derived prostate volume which has been shown to be very similar to prostate volume derived from transrectal ultrasound and is available for biopsy decision making.^19^

### 2.6 Statistical analysis

PHI and PSA density were evaluated for their ability to increase the accuracy in predicting GG2‐5 PCa beyond clinical data and PIRADS. Logistic regression models were used to generate receiver operating characteristic (ROC) curves and the area under the ROC curves (AUC), and their 95% confidence intervals were calculated. To evaluate the added accuracy of PHI with PIRADS, the following models were tested: 1) Log(PSA) + DRE, 2) Log(PSA) + DRE +Log(PHI), 3) Log(PSA) + DRE +PIRADS, 4) Log(PSA) + DRE +PIRADS + Log(PHI), and 5) Log(PSA) + DRE +PIRADS + Log(PHI) + Race (Black vs White). Models were tested for effect modification between Race and PHI and Race and PIRADS to determine if race modified the prediction accuracy of either tool. The PSA used in the models was the PSA that prompted the urologic referral. Similar models were created with PSA density. PSA, PSA density, and PHI were log base 10 transformed. The DRE was coded as a binary variable (ie, suspicious vs non‐suspicious). PIRADS was coded as an ordinal four‐level variable with PIRADS = 1‐2; = 3, and = 4 and = 5.

Thresholds of PHI and PSA density were generated, and the sensitivity, specificity, PPV, and NPV were compared for GG2‐5 PCa detection at each threshold and stratified by PIRADS scores as PIRADS = 1‐2, = 3, and = 4‐5. The highest GG reported for both the MRI‐targeted and systematic transrectal ultrasound‐guided biopsies was used as the outcome. Different PHI and PSA density thresholds were tested as a serial screening test after mpMRI to assess their impact on sensitivity, specificity, PPV, and NPV for GG2‐5 PCa detection.

Assuming a baseline AUC of 0.66 for PHI alone, we had 80% power to detect a 0.10 difference in AUC between a logistic regression model with PHI alone compared with a model with PHI and PIRADS. Using an alpha = .05, there was 83% power to detect a difference in accuracy using both tools in series with *n* = 143 men with correlated samples. All comparisons were two‐sided and *P*‐values <.05 indicated statistical significance. Statistical analysis was performed using SPSS 25 (IBM corporation 2017, United States) and MedCalc 19.0.5 (MedCalc Software, Belgium).

## 3 RESULTS

The study included 143 men, of which 78 identified as White and 65 as Black. Median age was 62.0 years, and 55 men (38.4%) had GG2‐5 PCa. Compared with White men, Black men had statistically higher medians for BMI (28.1 vs 26.6 kg/m^2^), PSA (7.2 vs 5.3 ng/mL), and PSA density (0.14 ng/mL/cm^3^ vs 0.10 ng/mL/cm^3^) and had greater frequencies of abnormal DRE (25.8% vs 9.0%) and GG2‐5 PCa (58.5% vs 21.8%) (see Table 1). There were no statistical differences between racial groups in age, PHI scores, family history of PCa, history of BPH/LUTS, marriage rates, and smoking history.

**TABLE 1:** Patient sociodemographic and clinical characteristics by racial group

Continuous variables	White	Black	*P*‐value^1^
	(n = 78)	(n = 65)	
	Median [IQR]	Median [IQR]	
Age, years	62.5 [55.0, 69.0]	61.0 [54.5, 70.0]	0.95
BMI, kg/m^2^	26.6 [24.4, 29.7]	28.1 [25.8, 31.4]	**0.02**
PSA, ng/mL	5.3 [3.7, 7.3]	7.2 [4.7, 10.9]	**0.002**
%free PSA	0.16 [0.11, 0.20]	0.11 [0.07, 0.18]	**0.004**
PSA density, ng/mL/cm^3^	0.10 [0.07, 0.16]	0.14 [0.09, 0.37]	**<0.001**
PHI	63.0 [40.4, 101.3]	60.9 [41.8, 98.2]	0.85

Categorical variables	n (%)	n (%)	***P*‐value^2^**
Family History of PCa	17/68 (25.0%)	18/59 (30.5%)	0.49
History of BPH/LUTS	16/78 (20.5%)	14/65 (21.5%)	0.88
Abnormal DRE	7/78 (9.0%)	16/62 (25.8%)	**0.008**
*PIRADS*			0.053
1‐2	15/78 (19.3%)	11/65 (16.9%)	
3	31/78 (39.7%)	16/65 (24.6%)	
4‐5	32/78 (41.0%)	38/65 (58.5%)	
Any PCa (GG ≥1)	29/78 (37.2%)	46/65 (70.8%)	**<0.001**
Clinically significant PCa (GG ≥2)	17/78 (21.8%)	38/65 (58.5%)	**<0.001**
Currently married	61/78 (78.2%)	37/65 (57.8%)	0.009
College completion	65/77 (84.4%)	31/64 (48.4%)	**<0.001**
Income < $60,000/year	9/77 (11.7%)	25/65 (39.1%)	**<0.001**
Smoking History, yes	29/78 (37.2%)	32/64 (50.0%)	0.17

Abbreviations: BMI, body mass index; BPH/LUTS, clinical diagnosis of benign prostatic hyperplasia; DRE, digital rectal exam; GG, Gleason grade group; PCa, prostate cancer; PHI, Prostate Health Index; PIRADS, Prostate Imaging Reporting and Data System score; PSA, prostate‐specific antigen.

^1^ Using Mann‐Whitney *U* test; ^2^Using *χ* ^2^ test.

PIRADS risk groups were as follows: 18.1% PIRADS 1‐2, 32.9% PIRADS 3, and 49.0% PIRADS 4‐5. As seen in Table 2, the rates of abnormal DREs and median PIRADS scores were significantly higher in men with GG2‐5 PCa compared to men with GG1 PCa and negative biopsies (*P* = .002 and *P* < .001, respectively). The proportion of men with GG2‐5 PCa was 23% in PIRADS 1‐2 men, 19% in PIRADS 3 men, 57% in PIRADS 4‐5 men. All six GG2‐5 PCas in the PIRADS 1‐2 group were found in Black men, even though Black men only constituted 42% of this group.

**TABLE 2:** Patient clinical characteristics by biopsy status

Continuous variables	Negative Biopsy	GG1 PCa	GG2‐5 PCa	*P*‐value^1^
	(n = 68)	(n = 20)	(n = 55)	
	Median [IQR]	Median [IQR]	Median [IQR]	
Age, years	63 [55, 69]	64 [58, 70]	61 [54, 69]	0.36
PSA, ng/mL	5.7 [3.8, 8.9]	5.1 [4.0, 7.0]	7.0 [4.7, 10.5]	0.75
%free PSA	0.16 [0.13, 0.22]	0.15 [0.87, 0.18]	0.095 [0.06, 0.14]	**<0.001**
PSA density, ng/ml/cm^3^	0.09 [0.06, 0.13]	0.11 [0.05, 0.14]	0.19 [0.12, 0.39]	**<0.001**
PHI	54.0 [30.2, 92.1]	56.2 [42.3, 95.3]	72.9 [56.3, 107.7]	**0.012**
*Categorical variables*	n (%)	n (%)	n (%)	***P*‐value^2^**
DRE abnormal	5/68 (7.3%)	2/20 (10.0%)	16/52 (29.1%)	**0.002**
*PIRADS*				**<0.001^3^**
1‐2	15/68 (22.1%)	5/20 (25.0%)	6/55 (10.9%)	
3	31/68 (45.6%)	7/20 (35.0%)	9/55 (16.4%)	
4‐5	22/68 (33.3%)	8/20 (40.0%)	40/55 (72.7%)	

Abbreviations: DRE, digital rectal exam; PCa, prostate cancer; PHI, Prostate Health Index; PIRADS, Prostate Imaging Reporting and Data System score; PSA, prostate‐specific antigen.

^1^ Using Kruskal‐Wallis test; ^2^Using χ2 test;^3^Overall distribution.

**FIGURE 1:**
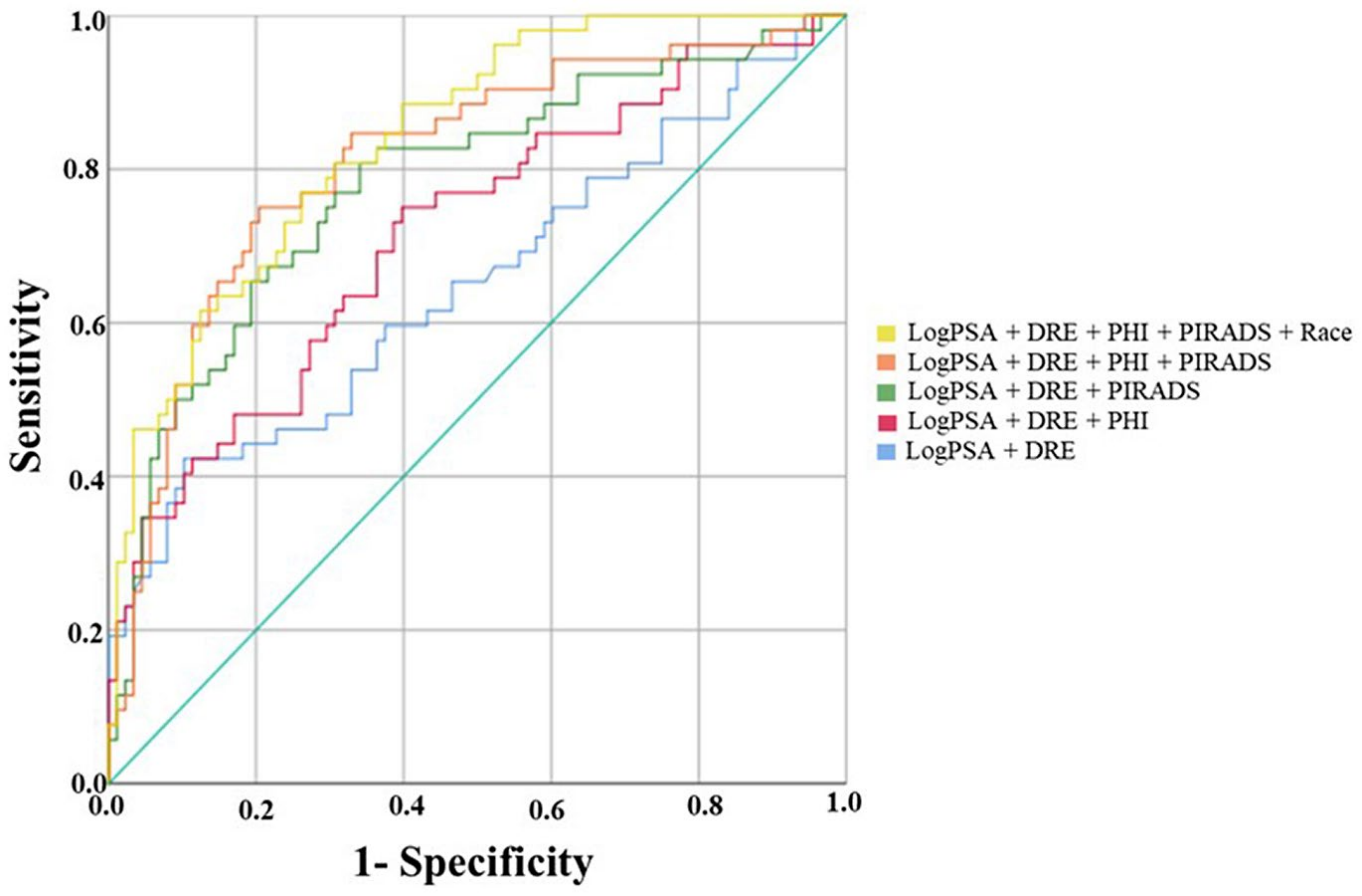
Area under the ROC curves (AUC) for detection of Gleason grade group 2‐5 prostate cancer from logistic regression models using PHI score

For the logistic regression models developed to identify GG2‐5 PCa, the base model included Log(PSA) + DRE and yielded an AUC of 0.66. The addition of PIRADS (AUC 0.78) or Log(PHI) (AUC 0.72) to the base model led to statistically significant higher AUCs compared with the base model alone (*P* = .001 and *P* = .03, respectively; Figure 1). When combining both PIRADS and Log(PHI) with the base model, the AUC increased to 0.81. Further adding race to such model yielded an AUC of 0.84, which was statistically better than the base model with PHI (*P* = .001) or PIRADS alone (*P* = .049). To assess for effect modification of race on the prediction accuracy of PIRADS and PHI, we tested the multiplicative interaction in the base model +PIRADS + Log(PHI) + Race. The interactions between PIRADS and Race (*P* = .08) and Log(PHI) and Race (*P* = .17) variables did not reach statistical significance.

**FIGURE 2:**
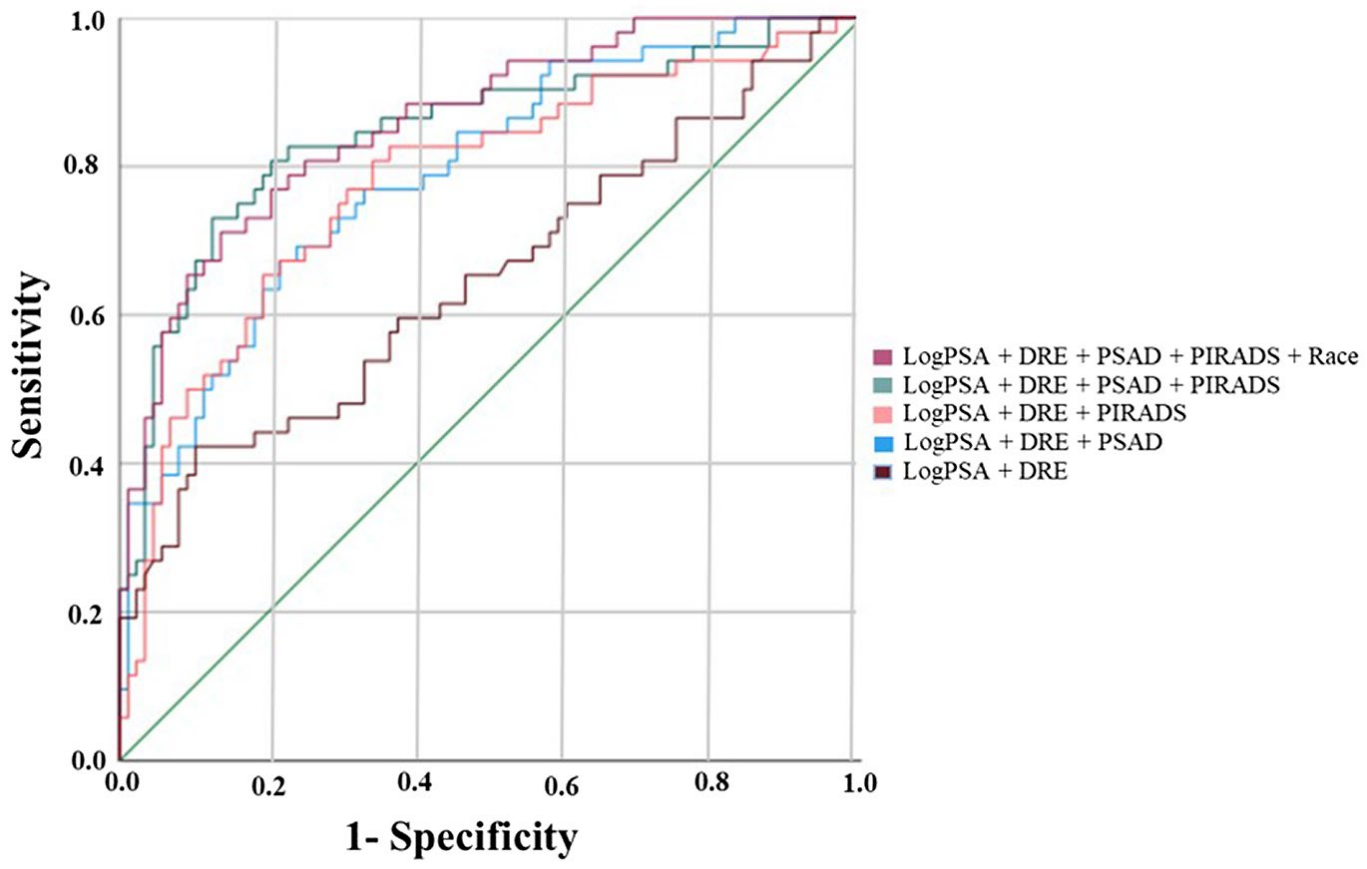
Area under the ROC curves (AUC) for detection of Gleason grade group 2‐5 prostate cancer from logistic regression models using PSA density

The models discussed were also constructed using PSA density instead of PHI scores. The base model +Log(PSA density) yielded an AUC of 0.79, which was higher than the base model alone (*P* = .002, Figure 2). The model with both PIRADS and Log(PSA density) increased the AUC to 0.85. The addition of race to the model resulted in an AUC of 0.86, which was statistically higher than the base model with Log(PSA density) (*P* = .04) or PIRADS alone (*P* = .005). The interaction between Log(PSA density) and Race (*P* = .42) was not statistically significant (Figure 2).

The last models were also constructed using both PSA density and PHI scores. The base model +Log(PHI) + Log(PSA density) had an AUC of 0.83 for the detection of GG2‐5 PCa, which was statistically higher than the base model +Log(PHI) alone (*P* = .002), but not for the Log(PSA density) model alone (*P* = .08). Overall, PSA density led to higher AUCs relative to PHI scores across all model iterations, yet these were not statistically significant.

Sensitivity and specificity analysis for PHI and PSA density were performed for PIRADS 1‐2, 3, and 4‐5 lesions. In men with a negative MRI (n = 26), that is, PIRADS 1‐2 scores, a PSA density ≥0.11ng/mL/cm^3^ detected 100% of the GG2‐5 PCa (n = 6) but subjected 30% of men with a negative MRI to biopsy (false positives). Meanwhile, a PHI ≥45 also detected 100% of GG2‐5 PCa in men with a PIRADS 1‐2 lesion but would subject 60% of men with negative MRIs to an unnecessary prostate biopsy.

In PIRADS 3 men, a PSA density threshold of ≥0.07 ng/mL/cm^3^ detected 88.9% of GG2‐5 PCa, while avoiding 32% of unnecessary biopsies. While a PHI score of ≥49 prevented unnecessary biopsies in 55.3% of men and missed zero cases of GG2‐5 PCa (see Table 3). This cutoff had a PPV of 34.6% and NPV of 100%. A combination of both PHI and PSA density thresholds was also calculated. If only men with PHI scores ≥49 and PSA density ≥0.07 were biopsied, one GG2‐5 PCa would be missed, making the sensitivity 88.9% but increasing the specificity to 71.1%. A sensitivity of 88.9% is achieved with a PHI threshold of ≥52, but the specificity (57.9%) is lower compared with the combination of both biomarkers.

**TABLE 3:** Sensitivity and specificity analysis for the detection of clinically significant prostate cancer by PHI and PSA density cutoffs in men with PIRADS 3 lesions (n = 47)

	Sensitivity	Specificity	PPV	NPV
*PHI*				
≥35	100.0%	26.3%	24.3%	100.0%
≥40	100.0%	36.8%	27.3%	100.0%
≥45	100.0%	50.0%	32.1%	100.0%
≥49	100.0%	55.3%	34.6%	100.0%
≥50	88.9%	55.3%	32.0%	95.5%
*PSA density*				
≥0.05	100%	7.9%	20.5%	100%
≥0.07	88.9%	31.6%	23.5%	92.3%
≥0.09	66.7%	42.1%	21.4%	84.2%
≥0.11	66.7%	60.5%	28.6%	88.5%

In PIRADS 4‐5 men, a sensitivity of 97.5% was achieved with a PHI score ≥27 and PSA density ≥.05 but they only avoided biopsies in 3.3% and 10% of men, respectively.

## 4 DISCUSSION

Our ROC analyses showed that PHI, PSA density, and PIRADS are independent predictors of GG2‐5 PCa in biopsy‐naïve men. Black race was an independent predictor of GG2‐5 PCa but did not modify the effect of PHI, PSA density, or PIRADS scores. Our current sample size precludes a thorough analysis of sensitivity and specificity by race.

PHI and PSA density resulted in improved detection of GG2‐5 PCa in PIRADS 1‐2 lesions but subjected a lot of men to unnecessary prostate biopsies. PHI and PSA density minimally increased the number of safely avoided biopsies in men with PIRADS 4‐5 lesions but missed too many GG2‐5 PCas. Interestingly, among PIRADS 1‐2 lesions, all six of the GG2‐5 PCas were from the Black participants, who only comprised only 42% of participants with PIRADS 1‐2 lesions. This suggests that the NPV of a negative MRI may be lower in Black men. However, this warrants validation in larger studies.

Although across all PIRADS categories PSA density yielded higher AUCs, in men with PIRADS 3, PHI proved to be more useful than PSA density in eliminating unnecessary biopsies. Since PIRADS 3 lesions are considered equivocal with 8%‐47% rates of GG2‐5 PCa, this is an area where an additional screening test used in series with adequate specificity would allow more men to avoid unnecessary biopsies.^11^, ^12^ PIRADS 3 lesions were present in 32.9% of participants and was the most frequently detected score on mpMRI. Our study demonstrated that when PHI is used as a serial test to PIRADS 3 lesions, a PHI threshold of ≥49 avoided unnecessary biopsies in 55% of men without missing any GG2‐5 PCa. Reducing the rate of unnecessary biopsy by half among PIRADS 3 lesions prevents avoidable biopsies in nearly one of seven men (13.7%) with a positive MRI (ie, PIRADS 3‐5 lesion). Additionally, we found that using a combination of PHI and PSA density scores might further decrease the number of unnecessary biopsies. If only men with PHI scores ≥49 and PSA density ≥0.07 were biopsied, 15% more men would be spared from an unnecessary biopsy with only one case of GG2‐5 PCa missed.

To date, no US studies have investigated the effect of PHI on prostate cancer detection in exclusively biopsy‐naïve patients undergoing biopsy following mpMRI. While PSA density was shown to be helpful in PIRADS 3 lesions in biopsy‐naïve patients, PHI has not been investigated explicitly within PIRADS 3 scores. A study by Gnanapragasam et al. assessed PHI in a cohort of men undergoing repeat biopsy and reported that adding PHI improved overall and GG2‐5 PCa detection determined by AUC, compared with mpMRI and PSA alone.^22^ In their cohort, a PHI threshold of ≥35 demonstrated a NPV of 0.97 for GG2‐5 PCa and spared 42% of men an unnecessary biopsy, while missing 5% of GG2‐5 PCa. However, this study did not report sensitivity, specificity, or NPV of PHI in PIRADS 3 lesions. Furthermore, their results were based on the older PIRADS version 1 criteria. Our study adds to existing knowledge by assessing the use of PHI in biopsy‐naïve men with equivocal PIRADS 3 lesions, as these patients have the most uncertainty regarding GG2‐5 PCa risk.

Certain limitations of our study should be noted. Our sample size in this study was relatively small, recruited from tertiary and publicly funded medical centers, and included only Black and White men, which may limit the generalizability of our results to men of other races. We were also not powered for race‐stratified analyses. Furthermore, we could have missed some men with GG2‐5 PCa by not directly targeting all PIRADS 1‐2 lesions at prostate biopsy. However, this is consistent with the current prostate biopsy standard of care.^26^ Our findings should be validated in a larger cohort with representation of other ethnic minorities.

## 5 CONCLUSION

PHI, PSA density, and mpMRI of the prostate are independent predictors that aid in the detection of GG2‐5 PCa in a biopsy‐naïve prostate biopsy cohort. PHI is particularly useful in men with equivocal PIRADS 3 lesions where it safely avoids over 50% of unnecessary biopsies and is superior to PSA density in this regard. Using both PHI and PSA density in series may further increase specificity and lead to fewer unnecessary biopsies, but further larger studies are warranted to determine the optimal threshold of each biomarker.

## Supporting information

Table S1

Table S2

## References

[1] Schröder FH , Hugosson J , Roobol MJ , Tammela TLJ , Ciatto S , Nelen V , et al. Prostate‐cancer mortality at 11 years of follow‐up. N Engl J Med. 2012;366(11):981–990. 22417251 10.1056/NEJMoa1113135 PMC6027585

[2] Tosoian J , Loeb S . PSA and beyond: the past, present, and future of investigative biomarkers for prostate cancer. Sci World J. 2010;10:1919–1931. 10.1100/tsw.2010.182 PMC5763794 20890581

[3] Grossman DC , Curry SJ , Owens DK , Bibbins‐Domingo K , Caughey AB , Davidson KW , et al. Screening for prostate cancer: US Preventive Services Task Force recommendation statement. JAMA. 2018;319(18):1901–1913. 29801017 10.1001/jama.2018.3710

[4] Schoots IG , Roobol MJ , Nieboer D , Bangma CH , Steyerberg EW , Hunink MM . Magnetic resonance imaging–targeted biopsy may enhance the diagnostic accuracy of significant prostate cancer detection compared to standard transrectal ultrasound‐guided biopsy: a systematic review and meta‐analysis. Eur Urol. 2015;68(3):438–450. 25480312 10.1016/j.eururo.2014.11.037

[5] de Rooij M , Hamoen EH , Fütterer JJ , Barentsz JO , Rovers MM . Accuracy of multiparametric MRI for prostate cancer detection: a meta‐analysis. Am J Roentgenol. 2014;202(2):343–351. 24450675 10.2214/AJR.13.11046

[6] Tan N , Margolis DJ , Lu DY , King KG , Huang J , Reiter RE , et al. Characteristics of detected and missed prostate cancer foci on 3‐T multiparametric MRI using an endorectal coil correlated with whole‐mount thin‐section histopathology. Am J Roentgenol. 2015;205(1):W87–W92. 26102423 10.2214/AJR.14.13285 PMC4898906

[7] Le JD , Tan N , Shkolyar E , Lu DY , Kwan L , Marks LS , et al. Multifocality and prostate cancer detection by multiparametric magnetic resonance imaging: correlation with whole‐mount histopathology. Eur Urol. 2015;67(3):569–576. 25257029 10.1016/j.eururo.2014.08.079

[8] Fütterer JJ , Briganti A , De Visschere P , Emberton M , Giannarini G , Kirkham A , et al. Can clinically significant prostate cancer be detected with multiparametric magnetic resonance imaging? A systematic review of the literature. Eur Urol. 2015;68(6):1045–1053. 25656808 10.1016/j.eururo.2015.01.013

[9] Zhang Z‐X , Yang J , Zhang C‐Z , Li K‐A , Quan Q‐M , Wang X‐F , et al. The value of magnetic resonance imaging in the detection of prostate cancer in patients with previous negative biopsies and elevated prostate‐specific antigen levels: a meta‐analysis. Acad Radiol. 2014;21(5):578–589. 24703470 10.1016/j.acra.2014.01.004

[10] Woo S , Suh CH , Kim SY , Cho JY , Kim SH . Diagnostic performance of prostate imaging reporting and data system version 2 for detection of prostate cancer: a systematic review and diagnostic meta‐analysis. Eur Urol. 2017;72(2):177–188. 28196723 10.1016/j.eururo.2017.01.042

[11] Westphalen AC , Fazel F , Nguyen H , Cabarrus M , Hanley‐Knutson K , Shinohara K , et al. Detection of clinically significant prostate cancer with PI‐RADS v2 scores, PSA density, and ADC values in regions with and without mpMRI visible lesions. Int Braz J Urol. 2019;45(4):713–723. 31136112 10.1590/S1677-5538.IBJU.2018.0768 PMC6837611

[12] Washino S , Okochi T , Saito K , Konishi T , Hirai M , Kobayashi Y , et al. Combination of prostate imaging reporting and data system (PI‐RADS) score and prostate‐specific antigen (PSA) density predicts biopsy outcome in prostate biopsy naïve patients. BJU Int. 2017;119(2):225–233. 26935594 10.1111/bju.13465

[13] Catalona WJ , Partin AW , Sanda MG , Wei JT , Klee GG , Bangma CH , et al. A multicenter study of [‐2] pro‐prostate specific antigen combined with prostate specific antigen and free prostate specific antigen for prostate cancer detection in the 2.0 to 10.0 ng/ml prostate specific antigen range. J Urol. 2011;185(5):1650–1655. 21419439 10.1016/j.juro.2010.12.032 PMC3140702

[14] De La Calle C , Patil D , Wei JT , Scherr DS , Sokoll L , Chan DW , et al. Multicenter evaluation of the prostate health index to detect aggressive prostate cancer in biopsy naive men. J Urol. 2015;194(1):65–72. 25636659 10.1016/j.juro.2015.01.091 PMC4696043

[15] Stephan C , Vincendeau S , Houlgatte A , Cammann H , Jung K , Semjonow A . Multicenter evaluation of [− 2] proprostate‐specific antigen and the prostate health index for detecting prostate cancer. Clin Chem. 2013;59(1):306–314. 23213080 10.1373/clinchem.2012.195784

[16] Loeb S , Sanda MG , Broyles DL , Shin SS , Bangma CH , Wei JT , et al. The prostate health index selectively identifies clinically significant prostate cancer. J Urol. 2015;193(4):1163–1169. 25463993 10.1016/j.juro.2014.10.121 PMC4404198

[17] Tosoian JJ , Druskin SC , Andreas D , Mullane P , Chappidi M , Joo S , et al. Use of the Prostate Health Index for detection of prostate cancer: results from a large academic practice. Prostate Cancer Prostatic Dis. 2017;20(2):228–233. 28117387 10.1038/pcan.2016.72 PMC5429201

[18] Verma A , St Onge J , Dhillon K , Chorneyko A . PSA density improves prediction of prostate cancer. Can J Urol. 2014;21(3):7312–7321. 24978363

[19] Hansen NL , Barrett T , Koo B , Doble A , Gnanapragasam V , Warren A , et al. The influence of prostate‐specific antigen density on positive and negative predictive values of multiparametric magnetic resonance imaging to detect Gleason score 7–10 prostate cancer in a repeat biopsy setting. BJU Int. 2017;119(5):724–730. 27488931 10.1111/bju.13619

[20] Hansen NL , Kesch C , Barrett T , Koo B , Radtke JP , Bonekamp D , et al. Multicentre evaluation of targeted and systematic biopsies using magnetic resonance and ultrasound image‐fusion guided transperineal prostate biopsy in patients with a previous negative biopsy. BJU Int. 2016. 10.1111/bju.13711 27862869

[21] Tapia MF , Labra A , Adlerstein I , Olivares JP , Schultz M , Silva C , et al. PSA density as a new approach for management of PIRADS 3 patients. Revista Chilena de Radiología. 2019;25(4):119–127.

[22] Gnanapragasam VJ , Burling K , George A , Stearn S , Warren A , Barrett T , et al. The Prostate Health Index adds predictive value to multi‐parametric MRI in detecting significant prostate cancers in a repeat biopsy population. Sci Rep. 2016;6(1):1–8. 27748407 10.1038/srep35364 PMC5066204

[23] Nettey OS , Walker AJ , Keeter MK , Singal A , Nugooru A , Martin IK , et al. Self‐reported Black race predicts significant prostate cancer independent of clinical setting and clinical and socioeconomic risk factors. Urol Oncol. 2018;36(11):501.e1–501.e8. 10.1016/j.urolonc.2018.06.011 PMC6214716 30236853

[24] Weinreb JC , Barentsz JO , Choyke PL , Cornud F , Haider MA , Macura KJ , et al. PI‐RADS prostate imaging–reporting and data system: 2015, version 2. Eur Urol. 2016;69(1):16–40. 26427566 10.1016/j.eururo.2015.08.052 PMC6467207

[25] Egevad L , Delahunt B , Srigley JR , Samaratunga H . International Society of Urological Pathology (ISUP) grading of prostate cancer—An ISUP consensus on contemporary grading. APMIS. 2016;124(6):433–435. 10.1111/apm.12533 27150257

[26] Sathianathen NJ , Konety BR , Soubra A , Metzger GJ , Spilseth B , Murugan P , et al. Which scores need a core? An evaluation of MR‐targeted biopsy yield by PIRADS score across different biopsy indications. Prostate Cancer Prostatic Dis. 2018;21(4):573–578. 30038389 10.1038/s41391-018-0065-6

