# Supplementary material for: Performance of prostate health index and PSA density in a diverse biopsy‐naïve cohort with mpMRI for detecting significant prostate cancer
Source: BJUI Compass. 2021 Jun 15;2(6):370–6. doi: 10.1002/bco2.91 (PMC8988695; doi:10.1002/bco2.91)
Supplement: Supplementary file 1 — Table S1 [file BCO2-2-370-s002.docx]

Supplementary Table 1. Area under the ROC curves (AUC) for detection of Gleason grade group 2-5 prostate cancer from logistic regression models using PHI score

| Base Model: log_10_PSA + DRE | **AUC**  **(95% CI)** | **p-value**  **(v. Model#1)** | **p-value**  **(v. Model#2: PHI)** | **p-value**  **(v. Model#3: PIRADS)** | **p-value**  **(v. Model#4: PIRADS + PHI)** |
| --- | --- | --- | --- | --- | --- |
| #1 – base model | 0.65  [0.55, 0.75] | --- |  |  |  |
| #2 –base + PHI | 0.72  [0.63, 0.81] | **0.03** | -- |  |  |
| #3 –base + PIRADS | 0.78  [0.70, 0.86] | **0.001** | 0.16 | -- |  |
| #4 – base + PIRADS + PHI | 0.81  [0.74, 0.89] | **<0.001** | **0.01** | 0.11 | **--** |
| #5 –base + PIRADS + PHI + Race | 0.84  [0.78, 0.90] | **<0.001** | **0.001** | **0.049** | 0.27 |
| AUC: area under the curve; DRE: digital rectal exam; PHI: Prostate Health Index. PIRADS: Prostate Imaging Reporting and Data System Score; PSA: prostate-specific antigen. Race was coded as Black vs. White. | | | | | |
