# Supplementary material for: Performance of prostate health index and PSA density in a diverse biopsy‐naïve cohort with mpMRI for detecting significant prostate cancer
Source: BJUI Compass. 2021 Jun 15;2(6):370–6. doi: 10.1002/bco2.91 (PMC8988695; doi:10.1002/bco2.91)
Supplement: Supplementary file 2 — Table S2 [file BCO2-2-370-s001.docx]

Supplementary Table 2. Area under the ROC curves (AUC) for detection of Gleason grade group 2-5 prostate cancer from logistic regression models using PSA density

| Base Model: log_10_PSA + DRE | **AUC**  **(95% CI)** | **p-value**  **(vs. Model#1)** | **p-value**  **(vs. Model#2: PSA Density)** | **p-value**  **(vs. Model#3: PIRADS)** | **p-value**  **(vs. Model#4: PIRADS + PSA Density)** |
| --- | --- | --- | --- | --- | --- |
| #1 – base model | 0.65  [0.55, 0.75] | --- |  |  |  |
| #2 –base + PSA Density | 0.79  [0.72, 0.87] | **0.002** | -- |  |  |
| #3 –base + PIRADS | 0.78  [0.70, 0.86] | **0.001** | 0.80 | -- |  |
| #4 – base + PIRADS + PSA Density | 0.85  [0.78, 0.92] | **<0.001** | 0.10 | **0.01** | **--** |
| #5 –base + PIRADS + PSA Density + Race | 0.86  [0.80, 0.92] | **<0.001** | **0.04** | **0.005** | 0.44 |
| AUC: Aurea under the Curve; DRE: digital rectal exam; Prostate Imaging Reporting and Data System Score (models as an ordinal variable ranging from 1-2, 3, 4, 5); PSA: prostate specific antigen. Race was coded as Black vs. White. | | | | | |
